# Low Molecular Weight Fucoidan Alleviates Cardiac Dysfunction in Diabetic Goto-Kakizaki Rats by Reducing Oxidative Stress and Cardiomyocyte Apoptosis

**DOI:** 10.1155/2014/420929

**Published:** 2014-11-30

**Authors:** Xinfeng Yu, Quanbin Zhang, Wentong Cui, Zheng Zeng, Wenzhe Yang, Chao Zhang, Hongwei Zhao, Weidong Gao, Xiaomin Wang, Dali Luo

**Affiliations:** ^1^Department of Pharmacology, School of Chemical Biology & Pharmaceutical Sciences, Capital Medical University, Youanmenwai Street, No. 10 Xitoutiao, Fengtai District, Beijing 100069, China; ^2^Institute of Oceanology, Chinese Academy of Sciences, Qingdao 266071, China; ^3^Department of Anesthesiology and Critical Care Medicine, Johns Hopkins University School of Medicine, Baltimore, MD 21287, USA; ^4^Department of Physiology, Capital Medical University, Beijing 100069, China

## Abstract

Diabetic cardiomyopathy (DCM) is characterized by cardiac dysfunction and cardiomyocyte apoptosis. Oxidative stress is suggested to be the major contributor to the development of DCM. This study was intended to evaluate the protective effect of low molecular weight fucoidan (LMWF) against cardiac dysfunction in diabetic rats. Type 2 diabetic goto-kakizaki rats were untreated or treated with LMWF (50 and 100 mg/kg/day) for three months. The establishment of DCM model and the effects of LMWF on cardiac function were evaluated by echocardiography and isolated heart perfusion. Ventricle staining with H-E or Sirius Red was performed to investigate the structural changes in myocardium. Functional evaluation demonstrated that LMWF has a beneficial effect on DCM by enhancing myocardial contractility and mitigating cardiac fibrosis. Additionally, LMWF exerted significant inhibitory effects on the reactive oxygen species production and myocyte apoptosis in diabetic hearts. The depressed activity of superoxide dismutase in diabetic heart was also improved by intervention with LMWF. Moreover, LMWF robustly inhibited the enhanced expression of protein kinase C *β*, an important contributor to oxidative stress, in diabetic heart and high glucose-treated cardiomyocytes. In conclusion, LMWF possesses a protective effect against DCM through ameliorations of PKC*β*-mediated oxidative stress and subsequent cardiomyocyte apoptosis in diabetes.

## 1. Introduction

Cardiac dysfunction is the hallmark of DCM, which is independent of the occurrence of coronary atherosclerosis or hypertension. It has been characterized by reduced velocity of contraction and relaxation and depressed myocardial contractility and compliance [1]. Myocardial fibrosis and hypertrophy are the primary structural changes observed in diabetic cardiomyopathy [2]. The diabetic myocyte is susceptible to cell apoptosis and necrosis, which is paralleled by fibroblast replacement, resulting in interstitial fibrosis mediated primarily by TGF-*β* [3]. Although the pathogenesis of DCM is multifactorial, several underlying mechanisms including alteration in cardiac energy metabolism [4], impaired Ca^2+^ homeostasis, [5, 6], and accumulation of ROS and cell death [7, 8] have been proposed. Of these, oxidative stress has been recognized as the important initiating factor and plays a pivotal role in the development of DCM [9–11].

Long-term hyperglycemia exerts the deleterious effects through overproduction of ROS that outweighs their degradation by antioxidant defense systems such as superoxide dismutase (SOD), catalase (CAT), heme oxygenase-1 (HO-1), and thioredoxin (Trx) [10, 11]. Oxidative stress also occurs in diabetic heart, resulting in cardiac dysfunction possibly by direct oxidative damage to cellular macromolecules, such as DNA and proteins [12, 13], disruption of Ca^2+^ homeostasis [14], and enhancement of cardiomyocyte apoptosis [15]. Furthermore, increasing evidence has implicated abnormal expression and activation of PKC*β* isoform in a broad array of diabetic complications [16]. PKC*β*-dependent activation of NAD(P)H oxidase, a prooxidant enzyme that serves as an important source of ROS production, has been demonstrated to be an essential mechanism responsible for the increased oxidative stress in diabetes [17]. Moreover, PKC*β* is preferentially overexpressed or activated in cardiomyocytes of rodents with diabetes; and inhibition of PKC*β* activity improves the cardiac function in diabetic rats [18, 19]. Therefore, increases in PKC*β* abundance and activity contribute significantly to the production of ROS and the development of DCM in diabetes.

Several antioxidants have been explored to ameliorate the morphological and functional changes of DCM [20–23]. However, although the targets at reducing ROS or increasing antioxidant activity represent valuable therapeutic approaches against DCM, it is still controversial for the application of pharmacologic antioxidants against diabetes in patients because of inadequate support for relieving pathological changes of DCM from clinical trials and also some adverse effects of these drugs [13, 24]. Fucoidans are a class of sulfated, fucose-rich polysaccharides extracted from brown seaweeds. LMWF (~7000 Dalton), extract from edible species of* Laminaria japonica* in Qingdao has been shown to have a spectrum of bioactivities such as antioxidation, anti-inflammation, and anticoagulation [25, 26]. We have previously demonstrated that LMWF protected against renal ischemia-reperfusion injury via inhibition of the MAPK signaling pathway [27], indicating LMWF has beneficial effects against oxidative stress injury. Furthermore, LMWF alleviates diabetic retinal neovascularization and damage via inhibition of vascular endothelial growth factor (VGEF) [28]. LMWF also induces endothelium-dependent vasodilation and protects vasoendothelial function in diabetic cardiovascular complications [29]. Moreover, the production of reproducible fucoidan fractions on a commercial scale and a lack of oral toxicity has made it practical for therapy by fucoidan [30]. In this study, we aimed to investigate whether LMWF exerts favorable actions on the development of DCM.

We compared LMWF-treated diabetic rats relative to untreated diabetic rats, and the results demonstrated that chronic treatment with LMWF produced a pronounced protective effect on DCM by improving cardiac contractile function, ameliorating the collagen deposition and improving the myocardium remodeling. In addition, the excessive PKC*β* expression and ROS production as well as the resultant cardiomyocyte apoptosis in diabetic rats were also attenuated by LMWF.

## 2. Materials and Methods

### 2.1. Drugs

As previously described [31], LMWF was produced from* Laminaria japonica *cultured in Qingdao and analyzed as follows: fucose content 29.5% (wt/wt); uronic acid content 7.5%; and sulfate content 30.1% [32]. The average molecular weight is about 7000 Da and LMWF was dissolved in physiological saline for animal treatment and in PBS for cell culture.

### 2.2. Characterization and Treatment of Diabetic Rats

All animal study protocols were approved by the Institutional Animal Research and Ethics Committee of Capital Medical University.

As previously described [29], twelve-week-old male GK rats and age-matched Wistar rats were purchased from SLAC Laboratory Animal Co., Ltd. (Shanghai, China) and reared in a standard experimental animal laboratory with normal food and water given ad libitum. Body weights were measured once a week, while blood glucose was examined once a month. Oral glucose tolerance test (OGTT) was also performed at the beginning of the experiment as previously described [29]. Blood glucose was measured from caudal vein with Accu-Chek Active glucometer (Roche Diagnostics, Indianapolis, IN, USA). All the animals were randomly divided into 5 groups with 12–15 rats in each group: (1) Wistar control rats (control group); (2) Wistar control rats treated with 100 mg/kg/day LMWF; (3) diabetic GK rats (DM group); (4) GK rats treated with LMWF (50 and 100 mg/kg/day, resp., referred to as F50 and F100 group). Diabetic GK rats were administered LMWF orally by gavage daily for consecutively 3 months and normal group and DM group were given equal amount of vehicle. Here it is needed to mention that all the physiological parameters of control animals administered with LMWF were similar to those of control group, so we only provide the data from control group.

### 2.3. Analysis of Left Ventricular Function by Echocardiography

Animals were subjected to isoflurane-based anesthesia and chest hair was removed by depilatory cream. M-mode echocardiography was performed using a high resolution ultrasound system (VisualSonics Vevo 770, Toronto, Canada) equipped with a high frequency transducer (frequency band 12–38 MHz). Left ventricular end-diastolic inner-dimension (LVIDd), diastolic left ventricular posterior/anterior wall thickness (LVPWd/LVAWd), and the corresponding systolic parameters of LVIDs and LVPWs/LVAWs were measured. The other parameters of cardiac output (CO), ejection fraction (EF), and fraction shortening (FS) were calculated by VisualSonics analysis software.

### 2.4. Isolated Heart Perfusion by Langendorff Method

The rats were anesthetized by intraperitoneal injection of 10% chloral hydrate (300 mg/kg). The heart was immediately removed and perfused at the constant pressure by the Langendorff method. Modified Krebs-Henseleit bicarbonate buffer (K-H buffer, mM: NaCl 118, KCl 4.7, CaCl_2 _2.0, MgSO_4_ 1.2, KH_2_PO_4_ 1.2, EDTA 0.5, NaHCO_3_ 25, glucose 11, pH 7.4, 37°C, 95% O_2_ + 5% CO_2_ gas mixture) was used as the perfusion buffer. A small balloon was inserted in the left ventricle through the left atrium to measure left ventricular pressure by a pressure transducer. The balloon was coupled to a microsyringe and balloon volume was adjusted to achieve a left ventricular end-diastolic pressure (LVEDP) of 5–10 mmHg at the beginning of the experiment. Peak left ventricular systolic pressure (LVP) and left ventricular developed pressure (LVDP) were calculated as LVP-LVEDP, and the maximum rate of pressure increase or decrease (±dp/dt) was determined by Powerlab software (AD Instruments, NSW, Australia).

### 2.5. Hematoxylin-Eosin (HE) and Sirius Red Staining

Paraffin-embedded left ventricle (LV) sections of 5 *μ*m in thickness were stained with Hematoxylin-Eosin and Sirius Red to identify the structural changes and measure myocardial fibrosis. The micrographs were taken by a microscope (Leica DM 4000B, Wetzlar, Germany). The extent of myocardial fibrosis was evaluated by the ratio of the positively stained fibrotic area to the whole area of the myocardium by Image J programme. The myocytes areas of LV cross sections were measured by Leica Qwin software.

### 2.6. RT-qPCR

Total RNA was extracted from the heart tissues of rats using TRIzol (Invitrogen, Carlsbad, CA) and reverse transcribed using SuperScript III First-Strand Synthesis System for reverse transcription- (RT-) PCR Kit (Invitrogen) according to the manufacturer's instructions. Real-time PCR was carried out in triplicate in an ABI 7500 real-time PCR system (Applied Biosystems) with a 20 *μ*L reaction mixture containing SYBR Green qPCR master mix and 300 nM fibronectin primers. The primer sequences for fibronectin were 5′-acaccatccaagtcctgagg-3′ and 5′-tctgatcggcatgaaccact-3′. The primer sequences for GAPDH were 5′-agacagccgcatcttcttgt-3′ and 5′-cttgccgtgggtagagtcat-3′. The thermal cycling was initiated by the polymerase activation step for 10 min at 95°C followed by 40 cycles of denaturation (95°C for 30 s) and annealing/extension (60°C for 1 min). The relative expression of fibronectin was normalized to GAPDH as an internal control and determined by a previously described method [33].

### 2.7. Terminal Deoxynucleotidyl Transferase-Mediated dUTP Nick End Labeling (TUNEL)

The formalin-fixed paraffin-embedded tissue sections were subjected to TUNEL assay according to the instructions in the reagent kits (Roche Molecular Biochemicals). Briefly, the sections were deparaffinized, rehydrated in graded alcohol series, and then placed in 3% hydrogen peroxide in methanol for 10 min at room temperature. Sections were then incubated with 20 *μ*g/mL proteinase K for 15 min. The sections were washed several times in PBS and then incubated with TdT-enzyme TUNEL reaction mixture at 37°C for 1 h; then POD (conjugated with horseradish peroxidase) was dropped on the slides. The 3,3-diaminobenzidine (DAB) was used as the substrate. The sections without TUNEL reaction mixture were used as negative control. These sections were visualized by a Leica inverted microscope at 400× magnification. TUNEL-positive cardiomyocytes were carefully evaluated under double-blind conditions. At least 3 visual fields were chosen from each section for analysis. The rate of apoptotic cell nuclei is defined as apoptotic positive cell nuclei/total cell nuclei in the field.

### 2.8. Activities of Antioxidant Enzymes and Malondialdehyde (MDA) Content in Heart Tissue

The samples of heart tissue were weighed and homogenized (1 : 10, w/v) in 0.9% sodium chloride (pH 7.4). The level of MDA and the activities of SOD and CAT in heart tissue were determined by colorimetric analysis using a UV-VIS spectrophotometer (UV-2550, Shimadzu, Japan). The procedures were performed and the results were analyzed by kits according to the company's instructions (Nanjing Jiancheng Bioengineering Institute, China).

### 2.9. Western Blot

The left ventricular tissue was lysed in RIPA buffer (20 mM Tris, pH 7.5, 150 mM NaCl, 1 mM EDTA, 1 mM EGTA, 1% Triton X-100, 1 mM Na_3_VO_4_, 1 mg/mL aprotinin, leupeptin and pepstatin, 1 mM Phenylmethylsulfonyl fluoride (PMSF)) and protein concentration was determined by bicinchoninic acid (BCA) protein assay (Pierce, Rockford, IL, USA). Equal amounts of protein were separated with a 12% SDS-PAGE and then transferred to PVDF membranes. After blocking with 5% defatting milk for 1 h at room temperature, the membranes were incubated with primary antibodies against PARP (Cell Signaling Technology), PKC*α*, PKC*β*1, PKC*β*2, Bax, and GAPDH (Santa Cruz Biotechnology, USA). Finally, the membranes were hybridized with horseradish peroxidase-conjugated secondary antibodies and blots were developed by an enhanced chemiluminescence detection kit (Pierce, Rockford, IL, USA). Band intensities were quantified using a densitometer analysis system (Quantity one software, Bio-Rad, PA, USA).

### 2.10. Intracellular ROS Detection

ROS was measured with membrane permeable dye 2′,7′-dichlorodihydrofluorescein diacetate molecule probes H_2_DCF-DA (D6883, Sigma), a nonfluorescent probe that is rapidly oxidized to the fluorescent probe in the presence of intracellular ROS. H9c2 cells were obtained from American Type Culture Collection (ATCC) and cultured in Dulbecco's Modified Eagle's Medium (DMEM) supplemented with 10% fetal bovine serum (FBS). H9c2 Cells were plated in 96-well plates at a density of 10^4^/well and incubated in normal glucose medium (NG, 5.5 mM D-glucose) or high glucose medium (HG, 25 mM D-glucose) with or without 20 *μ*g/mL LMWF. Normal glucose medium supplemented with 20 mM D-mannitol was used as an osmotic control. 100 *μ*M H_2_O_2_ was used as a positive control. After treatments for 24 h, the cell medium was removed and replaced with phosphate-buffered saline (PBS) containing 20 *μ*M H_2_DCF-DA for 45 min at 37°C in the dark and in a humidified atmosphere with 5% CO_2_. After washing with PBS, the fluorescence was measured in a microplate reader (FluoDia T70, NJ, USA) at excitation and emission wavelengths of 485 and 520 nm, respectively.

### 2.11. Cell Viability Assay

Cell viability assay was performed by a colorimetric 3-(4,5-dimethylthiazol-2-yl)-2,5-diphenyl tetrazolium bromide (MTT) assay. Briefly, the H9c2 cells were placed at a density of 5 × 10^3^ cells/well in 96-well plates. The cells were incubated in 100 *μ*L normal glucose medium (NG, 5.5 mM D-glucose) or high glucose medium (HG, 25 mM D-glucose) with or without 20 *μ*g/mL LMWF for 72 h. Normal glucose medium supplemented with 20 mM D-mannitol was used as an osmotic control. After treatment with 20 *μ*L MTT (5 mg/mL) for another 4 h, the solution was discarded and 150 *μ*L DMSO was added to each well. Absorbance was measured with a microplate reader (Bio-Rad Model 680, PA, USA) at 570 nm. We calculated the cell viability using the formula: Cell viability (%) = (OD_treatment  group_ − OD_blank_)/(OD_control  group_ − OD_blank_) × 100%.

### 2.12. Statistical Analysis

Data are presented as mean ± SEM unless otherwise stated. Data were evaluated by analysis of variance (ANOVA) with SPSS 16.0 software to show differences between the groups. A value of *P* < 0.05 was considered as statistically significant.

## 3. Results

### 3.1. LMWF Mitigates Left Ventricular Dysfunction in Diabetic Rats

As a model of spontaneous nonobese type 2 diabetes, GK rats were confirmed to be hyperglycemic throughout this experiment. All the diabetic GK rats at 13-14 week old showed greater hyperglycemic responses after oral administration of glucose in OGTT before drug intervention. After treatment of LMWF for 3 months, the level of blood glucose in diabetic groups was significantly higher than that of normal control (16.2 ± 3.9 versus 6.5 ± 0.4 mM, *P* < 0.05), while LMWF at the doses of 50 and 100 mg/kg/day did not improve the hyperglycemia in diabetic rats and the blood levels in F50 and F100 groups were 18.2 ± 4.2 mM and 17.7 ± 4.5 mM, respectively. It appears that LMWF has no effects on impaired glucose metabolism; however, it does protect against diabetes-induced hypertension and hyperlipidemia [29].

As echocardiographic parameters showed in Table 1, GK rats at the age of 6 months developed obvious cardiac dysfunction, demonstrated by substantially impaired cardiac output (CO), ejection fraction (EF), and fraction shortening (FS). After administration of LMWF (100 mg/kg/day) for 3 months, these parameters in diabetic animals were significantly improved. The EF in diabetic group was significantly reduced compared with that of control group (52.63 ± 2.76% versus 73.42 ± 2.20, *P* < 0.01), which can be robustly improved by the treatment of LMWF (64.34 ± 1.86% versus 52.63 ± 2.76%, *P* < 0.01), indicating a potential protective effect of LMWF on cardiac performance in vivo.

**Figure 1:**
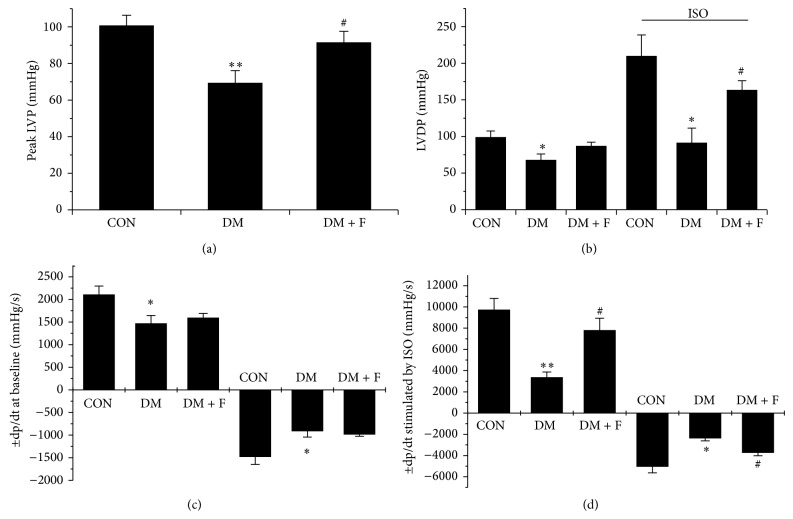
Influence of LMWF administration on myocardial contractility of diabetic GK rats determined in isolated perfused hearts. Peak left ventricular pressure (LVP) was shown in (a). Left ventricular developed pressures (LVDP) at both baseline levels and stimulated by isoprenaline (ISO, 10^−5 ^M) were shown in (b). The maximum rates of contraction and relaxation of myocardium (±dp/dt) at both baseline levels and in response to ISO stimulation were shown in ((c), (d)). *N* = 5–7 for each group, ^*^ *P* < 0.05, ^**^ *P* < 0.01, versus control group; ^#^ *P* < 0.05 versus DM group.

To confirm the protective effect of LMWF on left ventricular dysfunction, we further performed heart perfusion by Langendorff system to evaluate left ventricular function at both baseline and isoprenaline- (ISO-) stimulated levels. Peak left ventricular pressure (LVP), a measurement of left ventricular contractility, was significantly reduced in diabetic rats when compared with that of control rats. Treatment of diabetic rats with LMWF improved the cardiac contractility to levels comparable to controls (*P* < 0.05, Figure 1(a)). Moreover, animals in DM group also displayed significant reduction in left ventricular developed pressure (LVDP) and the maximum rate of contraction and relaxation (±dp/dt) when compared with those of control group at basal level. Although LMWF improved the above parameters in diabetic rats, there was no statistical significance (*P* > 0.05) (Figures 1(b) and 1(c)). However, when ISO, a nonselective beta-adrenergic agonist that induces positive chronotropic, dromotropic, and inotropic effects, was employed to mimic the activation of cardiac sympathetic nerve system, diabetic rats showed a prominent depression of LVDP and the maximum rate of contraction and relaxation (±dp/dt) due to DCM compared with those of normal rats. LMWF could significantly improve the dysfunction of cardiac contractility and compliance in response to ISO in diabetic rats (Figures 1(b) and 1(d)), suggesting that LMWF treatment can improve the working state or functional performance of diabetic hearts.

### 3.2. LMWF Reduces Interstitial Fibrosis

**Figure 2:**
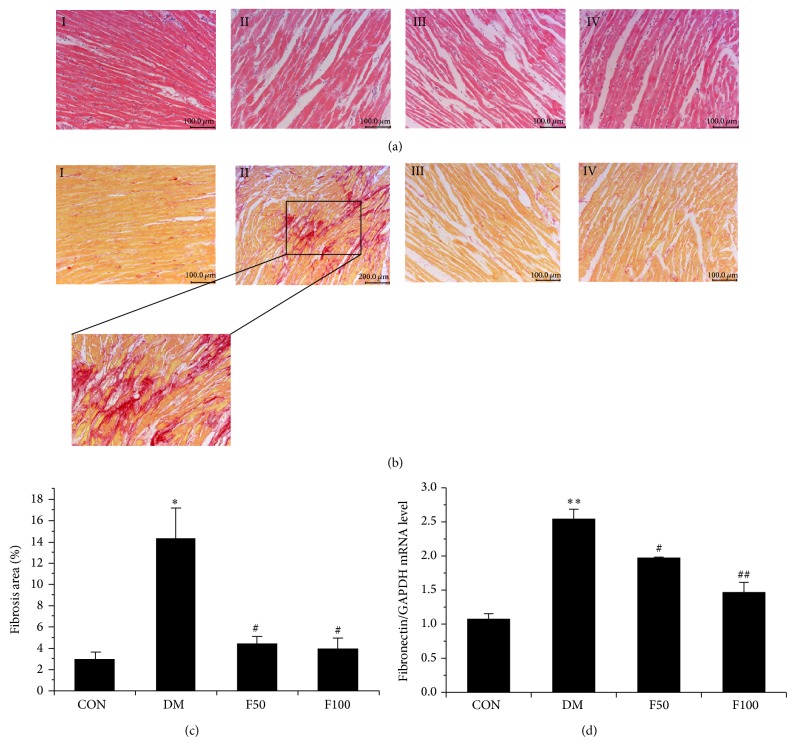
LMWF attenuated pathological changes in the hearts of diabetic GK rats. Representative micrographs of myocardial tissue sections stained with Hematoxylin and Eosin (a) and Sirius Red (b) for each group (*N* = 3, scale bar = 100 *μ*m). I represents control group, II represents DM group, and III and IV represent diabetic rats administered with 50 and 100 mg/kg/day LMWF, respectively. The black lined-box (in (b)) shows a high-magnification micrograph of Sirius Red-stained sections. Myocardial fibrosis was stained in red. (c) Fibrosis area was measured and statistical analysis was performed in different groups. (d) The level of fibronectin mRNA expression in heart tissues of each group was determined by real-time PCR, and the statistical analysis of fibronectin mRNA normalized to the housekeeping gene GAPDH was shown (*N* = 3). ^*^ *P* < 0.05, ^**^ *P* < 0.01 versus control group; ^#^ *P* < 0.05, ^##^ *P* < 0.01 versus DM group.

**Figure 3:**
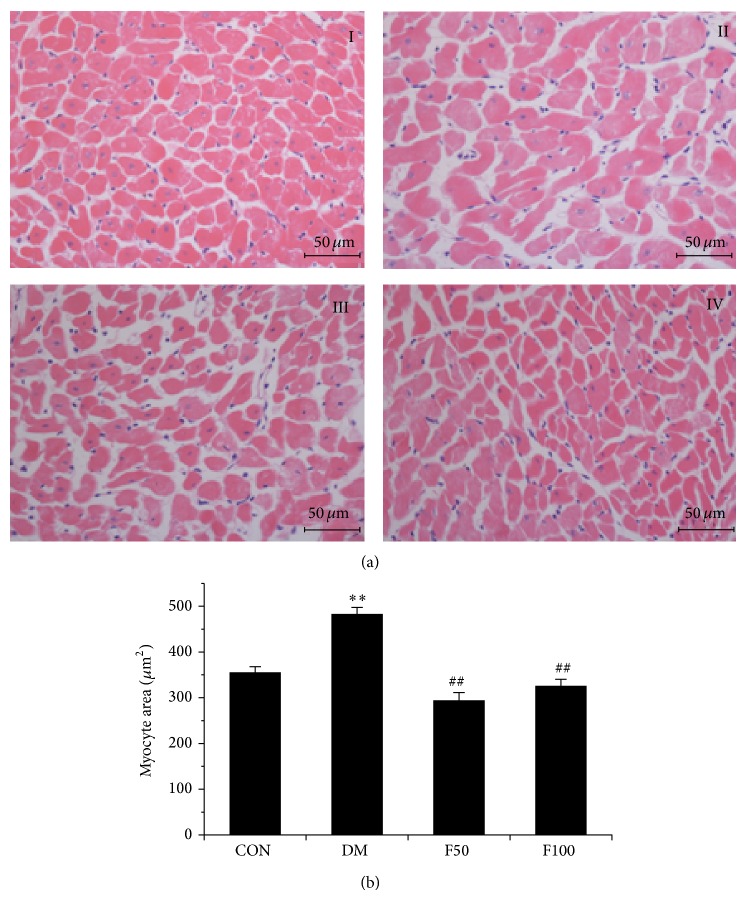
LMWF improved structural changes of myocardial tissue of diabetic GK rats. ((a), (b)) Representative micrographs of LV cross sections and myocyte cross-sectional area was measured in different groups and statistical analysis was performed (*N* = 3, scale bar = 50 *μ*m). I represents control group, II represents DM group, and III and IV represent diabetic rats administered with 50 and 100 mg/kg/day LMWF, respectively. ^**^ *P* < 0.01 versus control group; ^##^ *P* < 0.01 versus DM group.

It is well accepted that DCM was associated with a significant increase in myocardial fibrosis. Collagen content has been suggested to decisively influence the remodeling and mechanic properties of myocardium [34]. In order to explore whether long-term administration of LMWF has a beneficial impact on the structural changes of myocardium, we analyzed paraffin-embedded tissue sections by HE and Sirius Red staining. It revealed that obvious fibrosis and widening of the interstitial space were seen in hearts of diabetic rats compared with those in control group (Figures 2(a) and 2(b)). Moreover, compared with normal heart, interstitial and perivascular fibrosis was obviously accumulated in diabetic myocardium but was not prominently seen in diabetes rats treated with LMWF (Figures 2(b) and 2(c)). Cardiac fibrosis was further confirmed in diabetic myocardium by determining the expression of fibronectin, an extracellular matrix glycoprotein that plays an important role in abnormal cardiac muscle function. As shown in Figure 2(d), the mRNA level of fibronectin was significantly increased in the heart of GK rats compared to control rats (*P* < 0.05). Treatment with LMWF (50 or 100 mg/kg/day) significantly reduced the expressions of fibronectin when compared with diabetic group (*P* < 0.05). In the magnified micrographs, myocyte areas of LV cross sections were significantly increased in diabetic rats compared with those of control rats (Figure 3(a)). Further statistical analysis of the myocyte hypertrophy occurred in diabetic hearts demonstrated that LMWF at concentrations of 50 and 100 mg/kg/day significantly reduced the increased myocyte size (Figure 3(b), *P* < 0.05).

### 3.3. LMWF Inhibits Diabetes-Induced Cardiomyocyte Apoptosis

**Figure 4:**
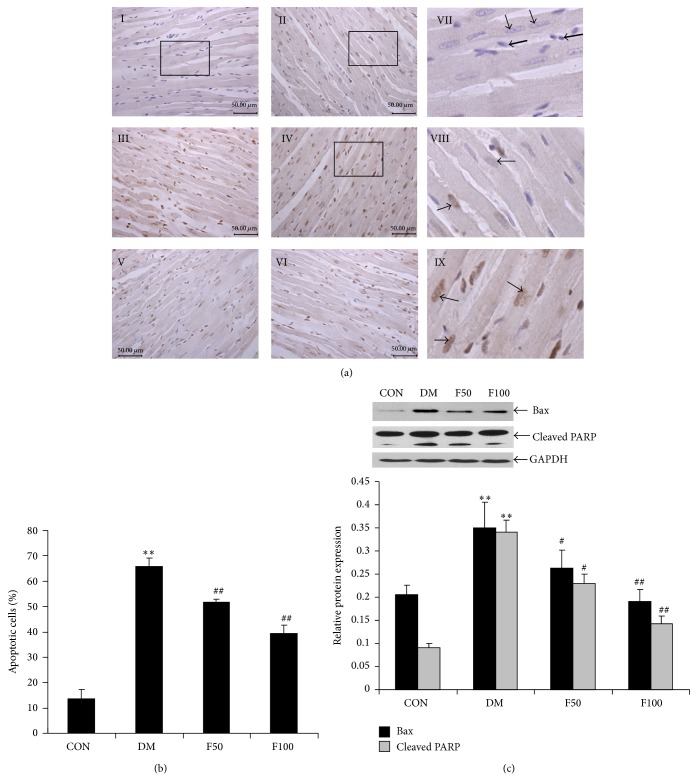
LMWF reduced apoptosis in the cardiac tissue of diabetic GK rats. (a) Representative micrographs of the TUNEL assay. I represents negative control group without TUNEL reaction mixture, II represents control group, III and IV represent DM group, and V and VI represent diabetic rats administered with 50 and 100 mg/kg/day LMWF, respectively (scale bar = 50 *μ*m, *N* = 3 in each group). The black lined-boxes (in I, II, and IV) were shown by high-magnification micrographs of VII, VIII, and IX, respectively. Bold arrows show the nuclei of blood cells while thin arrows show the nuclei of cardiomyocytes. (b) Statistical analysis of the percentage of apoptotic cells in each group by randomly selected 9 fields from each micrograph. (c) Western blots showing the expression of Bax and PARP in heart tissues of each group. The densitometry value of Bax and cleaved PARP was normalized to that of GAPDH and data are presented as the mean ± SEM. The statistical analysis was performed. ^**^ *P* < 0.01 versus control group; ^#^ *P* < 0.05, ^##^ *P* < 0.01 versus DM group. *N* = 9 in each group.

Myocardial cell death plays a critical role in the pathogenesis of DCM due to the loss of terminally differentiated cardiac myocytes, and inhibition of apoptosis by antioxidants results in a significant prevention of diabetic cardiotoxicity [35]. Here, as shown in Figure 4(a), the nuclei of apoptotic cardiomyocytes were stained in brown in contrast with blue staining of negative control by TUNEL assay. To exclude the disturbance of false positivity of blood cells, we only evaluate the apoptosis by counting the number of nuclei of cardiomyocytes which have larger nuclei than blood cells. Noticeably, the nuclei of apoptotic cardiomyocytes stained in brown in DM group (Figure 4(a), panels III and IV) were with higher intensity and larger proportion than those of control group (panel II); LMWF (50 or 100 mg/kg/day) treatment significantly attenuated the DNA fragmentation of diabetic cardiomyocytes (panels V and VI, and Figure 4(b), *P* < 0.05). Furthermore, Western blot was performed to detect the expression of apoptotic proteins. Bax is a mitochondrial protein that promotes cell apoptosis. PARP, a nuclear poly (ADP-ribose) polymerase, is involved in DNA repair in response to environmental stress and the cleavage of PARP serves as a marker of cells undergoing apoptosis. As shown in Figure 4(c), the expression of both Bax and cleaved PARP was enhanced significantly in DM group compared with that in control group (*P* < 0.05). Similarly, LMWF treatment inhibited the increase of Bax and PARP cleavage in cardiac tissue of diabetic rats.

### 3.4. LMWF Inhibits Oxidative Stress by Regulation of Antioxidant Enzymes and PKC Expression

**Figure 5:**
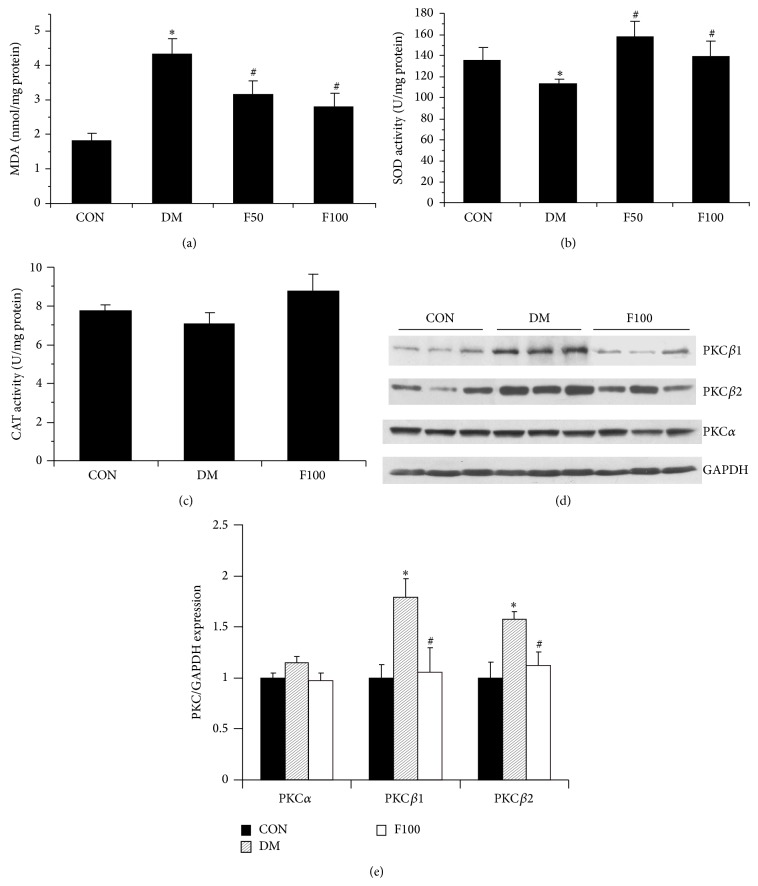
LMWF reduced oxidative stress in diabetic GK rats. Administration with 50 and 100 mg/kg/day LMWF decreased MDA content (a) and increased SOD activity (b) in cardiac tissues of diabetic rats. (c) Administration with 100 mg/kg/day LMWF has no significant effect on the CAT activity of cardiac tissue in diabetic rats. ^*^ *P* < 0.05 versus control group, ^#^ *P* < 0.05 versus DM group. *N* = 8–10 in each group. (d) Western blots were performed to determine the expression of PKC*β*1, PKC*β*2, and PKC*α* in cardiac tissues of each group. (e) The densitometry value of PKC was normalized to that of GAPDH and data are presented as the mean ± SEM. The statistical analysis was performed. ^*^ *P* < 0.05 versus control group, ^#^ *P* < 0.05 versus DM group. *N* = 9 in each group.

Hyperglycemia produces damaging effects through ROS accumulation in the heart. Oxidative stress occurs through the overproduction of ROS or the impaired elimination by antioxidant enzymes. In order to explore the cardioprotective mechanism of LMWF, the level of lipid peroxides and activities of antioxidant enzymes (SOD and CAT) were determined. As shown in Figure 5, there was an increased accumulation of lipid peroxides in diabetic rats as indicated by the higher level of MDA. Consistently, the enzymatic activity of antioxidant SOD was decreased significantly compared with that in controls (*P* < 0.05). The treatment of LMWF enhanced the SOD activity and inhibited the MDA content in diabetic rats (*P* < 0.05 versus DM group). CAT also displayed a similar trend in diabetic rats but with no statistical significance (*P* > 0.05).

In addition to the changes in antioxidant enzymes, abnormal activation of PKC*β* resulting from increased formation of diacylglycerol (DAG) by hyperglycemia, has been proposed as an important contributor to the production of ROS in diabetic cardiomyocytes in a positive feedback manner [18]. In order to explore whether LMWF has any effect on the altered expression of PKC*β*, the expression of PKC in cardiac tissues from each group was detected by Western blotting. As shown in Figures 5(d) and 5(e), isotypes of PKC*β*_1_ and PKC*β*_2_ were both significantly overexpressed in diabetic hearts, and this diabetes-associated specific change could be attenuated by LMWF intervention in diabetic rats.

**Figure 6:**
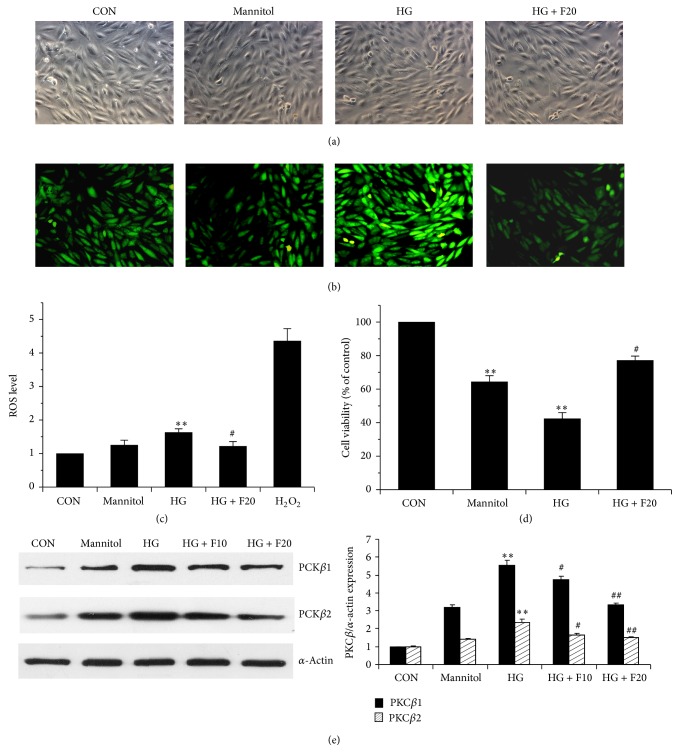
LMWF directly inhibited PKC*β*-mediated ROS production in H9c2 cells induced by high glucose. ((a), (b)) H9c2 cells were incubated in normal glucose (NG, 5.5 mM D-glucose) and treated with high glucose (HG, 25 mM D-glucose) without or with LMWF (20 *μ*g/mL) treatment for 24 h. D-mannitol (20 mM) was used as an osmotic control. Then cells were incubated with 20 *μ*M H_2_DCF-DA at 37°C for 45 min. After washing with PBS, fluorescence intensity was examined by fluorescence microscope (excitation wavelength = 485 nm, exposure time = 10 ms). The micrographs were taken by both light and fluorescence microscopes in the same fields. (c) H9c2 cells were seeded in 96-well microplates and subjected to the same treatment as above to detect ROS production. H_2_O_2_ was used as a positive control. (d) H9c2 cells were seeded in 96-well plates and subjected to the same treatment as above, after 72 h, cell viability was determined by MTT assay. (e) H9c2 cells were seeded in 6-well plates and were treated with HG (25 mM D-glucose) without or with LMWF (10, 20 *μ*g/mL) treatment for 48 h. Then cells were harvested and protein lysates were subjected to Western blot analysis of the expression of PKC*β*1 and PKC*β*2 relative to the expression of *α*-actin. The densitometry value of PKC was normalized to that of *α*-actin and statistical analysis was performed. Data are presented as the mean ± SEM. ^**^ *P* < 0.01 versus control or D-mannitol group, ^#^ *P* < 0.05, ^##^ *P* < 0.01 versus HG group.

Finally, the inhibitory effect of LMWF on the production of ROS, PKC*β* overexpression, and cell apoptosis were examined in cultured rat cardiomyocytes H9c2 that were challenged with high glucose (25 mM D-glucose). As shown in Figure 6, LMWF at concentration of 20 *μ*g/mL significantly attenuated high glucose-induced PKC*β* overexpression and ROS production in H9c2 cells. In addition, the high glucose-induced cell death was also suppressed by 20 *μ*g/mL LMWF determined by the MTT assay.

## 4. Discussion

The present study demonstrates that prolonged treatment of type 2 diabetic GK rats with LMWF produces a protective effect against cardiac dysfunction. This judgment is mainly based on the following supporting data: (1) LMWF improves cardiac dysfunction by increasing the contraction and compliance of myocardium evaluated by echocardiography and isolated heart perfusion (Table 1, Figure 1); (2) LMWF ameliorates the cardiac remodeling by reducing myocardium fibrosis and hypertrophy (Figures 2 and 3); (3) LMWF reduces oxidative stress and hence cardiomyocyte apoptosis by improving the antioxidant enzymes and attenuation of PKC*β*-mediated ROS production (Figures 4–6).

DCM is one of the serious complications of diabetes and characterized by early-onset diastolic and late-onset systolic dysfunctions as well as cardiac remodeling. It is well known that 90% diabetes patients around the world are inflicted with type 2 diabetes and most of them are susceptible to cardiac dysfunction. Spontaneous diabetic GK rats represent type 2 diabetic phenotype and may develop DCM after several weeks [36]; thus we adopted GK rats to explore the effect of LMWF on cardiac dysfunction. Cardiac fibrosis and cardiomyocyte apoptosis are important characteristics of DCM and responsible for cardiac remodeling [37, 38]. The present study demonstrates that GK rats developed marked cardiac dysfunction as shown by echocardiographic parameters and isolated heart perfusion as well as increased myocardium fibrosis and hypertrophy. Treatment with LMWF plays a crucial role in protecting against cardiac dysfunction not only by ameliorating the blood pressure and lipids metabolism, but also by preventing oxidative stress mediated myocardial injury and remodeling.

Oxidative stress, an imbalance between endogenous ROS and antioxidant systems, is involved in the development of diabetes complications, both microvascular and cardiovascular [7, 8, 39]. The metabolic abnormalities and insulin resistance of diabetes cause mitochondrial ROS overproduction from free fatty acids in the cardiomyocytes. On the one hand, ROS alters Ca^2+^-handling protein such as RyR, SERCA, and phospholamban (PLB) and their abnormalities in return impair calcium homeostasis [14, 40], resulting in inotropic defects and heart dysfunction. On the other hand, oxidative stress has been shown to be linked with increased cardiomyocyte apoptosis in diabetes, which promotes cardiac remodeling and contributes to DCM [41–43]. MDA, an end-product of lipid peroxidation, has been shown to be mutagenic by forming DNA adducts, representing the level of free radicals that can damage fatty acids [44]. In this study, we found that MDA was significantly increased in diabetic cardiac tissues, which implied that hyperglycemia can induce peroxidation of lipids and progressively increase oxidative stress in the hearts of diabetic GK rats. In addition, decreased levels of antioxidant enzymes in the diabetic rats were also found to make the cardiac tissues more susceptible to oxidative damage [45]. Here, the antioxidant enzyme SOD is markedly reduced in diabetic heart, and another antioxidant enzyme CAT displayed the similar trend although there is no statistical significance. Thus, these results were consistent with previous studies that increased superoxide production and lipid peroxidation and decreased activities of antioxidant enzymes (GPx, SOD, and CAT) exist in type 2 diabetes [46, 47]. LMWF could reduce the production of MDA and enhance the activity of SOD in GK rats.

In addition, LMWF also mitigated the enhanced expression of PKC*β* in diabetic rats. Alteration in PKC*β* level or activity has been implicated in diabetic damage [48]. PKC*β* upregulation in diabetes forms a positive feedback loop to increase diabetes-induced oxidative stress [49], and PKC*β*2 activation is associated with increased expression of connective tissue growth factor which could induce cardiac fibrosis in diabetes [15]. A potential antioxidant N-acetylcysteine has been demonstrated to attenuate the PKC*β*2 overexpression and cardiac remodeling [19]. In this study, we also found that LMWF significantly inhibited the overexpression of PKC*β*. In addition, in rat ventricular myocytes H9c2 cells that were exposed to high glucose, the increased PKC*β* expression, ROS production, and cell death induced by high glucose were significantly attenuated by LMWF treatment, suggesting the beneficial effect of LMWF in the harmful chain of PKC/ROS/apoptosis triggered by high glucose.

To date, a number of chemicals and agents have been explored to reduce the cardiac cell apoptosis and oxidative stress in animal models of diabetes [20, 21, 23], but few can be finally developed into clinical uses because of its insufficient effectiveness and substantial adverse effects in human [13, 24]. LMWF, an extract from natural occurring brown algae, has been shown to have favorable toxic profile [50, 51]. Thus, it is possible for development of LMWF in final stage based on the efficacy and safety of LMWF in the prevention and treatment of diabetic complication.

In summary, the present study provides the first evidence for the protective effect of LMWF on the development of DCM. LMWF inhibits oxidative stress and resultant cardiomyocyte apoptosis in diabetic heart by potentiating the antioxidant enzymatic activity and inhibiting PKC*β*-dependent ROS production. In light of its efficacy and safety, LMWF may serve as a potential therapeutic drug for prevention and treatment of DCM.

## Figures and Tables

**Table 1:** Echocardiographic parameters of rats from different groups.

Parameters	CON (*N* = 6)	DM (*N* = 8)	F100 (*N* = 6)
LVID;d (mm)	8.64 ± 0.24	8.68 ± 0.19	7.82 ± 0.35^#^
LVPW;d (mm)	1.76 ± 0.13	1.67 ± 0.13	1.67 ± 0.11
LVID;s (mm)	4.80 ± 0.18	6.20 ± 0.19^**^	4.96 ± 0.23^##^
LVPW;s (mm)	2.99 ± 0.19	2.08 ± 0.091^**^	2.35 ± 0.18
LVAW;d (mm)	1.71 ± 0.12	1.37 ± 0.066^*^	1.39 ± 0.071
LVAW;s (mm)	1.97 ± 0.11	1.67 ± 0.064^*^	1.74 ± 0.11
LV Vol;d (*μ*l)	411.69 ± 24.99	415.53 ± 19.63	398.62 ± 32.58
LV Vol;s (*μ*l)	108.51 ± 9.84	196.01 ± 13.93^**^	108.21 ± 14.27^##^
CO (*µ*l)	303.17 ± 22.87	219.52 ± 14.95^**^	290.41 ± 31.79^#^
EF (%)	73.42 ± 2.20	52.63 ± 2.76^**^	64.34 ± 1.86^##^
FS (%)	44.41 ± 1.91	28.56 ± 1.80^**^	36.54 ± 1.43^##^
LV mass/body weight (%)	0.20 ± 0.024	0.24 ± 0.030^*^	0.23 ± 0.015

LVIDd(s): left ventricular end-diastolic (systolic) inner-dimension; LVPWd(s): diastolic (systolic) left ventricular posterior wall thickness; LVAWd(s): diastolic (systolic) left ventricular anterior wall thickness; LV Vol d(s): left ventricular diastolic (systolic) volume; CO: cardiac output; EF: ejection fraction; FS: fraction shortening. Each value is mean ± SEM for 6–8 rats in each group. ^*^ *P* < 0.05 and ^**^ *P* < 0.01 versus control, respectively. ^#^ *P* < 0.05 and ^##^ *P* < 0.01 versus diabetes, respectively.

## References

[1] Poornima I. G., Parikh P., Shannon R. P. (2006). Diabetic cardiomyopathy: the search for a unifying hypothesis. *Circulation Research*.

[2] Goyal B. R., Mehta A. A. (2013). Diabetic cardiomyopathy: pathophysiological mechanisms and cardiac dysfuntion. *Human & Experimental Toxicology*.

[3] Booz G. W., Baker K. M. (1995). Molecular signalling mechanisms controlling growth and function of cardiac fibroblasts. *Cardiovascular Research*.

[4] An D., Rodrigues B. (2006). Role of changes in cardiac metabolism in development of diabetic cardiomyopathy. *American Journal of Physiology—Heart and Circulatory Physiology*.

[5] Suarez J., Scott B., Dillmann W. H. (2008). Conditional increase in SERCA2a protein is able to reverse contractile dysfunction and abnormal calcium flux in established diabetic cardiomyopathy. *American Journal of Physiology—Regulatory Integrative and Comparative Physiology*.

[6] Turan B., Vassort G. (2011). Ryanodine receptor: a new therapeutic target to control diabetic cardiomyopathy. *Antioxidants and Redox Signaling*.

[7] Giacco F., Brownlee M. (2010). Oxidative stress and diabetic complications. *Circulation Research*.

[8] Khullar M., Al-Shudiefat A. A.-R. S., Ludke A., Binepal G., Singal P. K. (2010). Oxidative stress: a key contributor to diabetic cardiomyopathy. *Canadian Journal of Physiology and Pharmacology*.

[9] Aksakal E., Akaras N., Kurt M., Tanboga I. H., Halici Z., Odabasoglu F., Bakirci E. M., Unal B. (2011). The role of oxidative stress in diabetic cardiomyopathy: an experimental study. *European Review for Medical and Pharmacological Sciences*.

[10] Halliwell B. (2007). Biochemistry of oxidative stress. *Biochemical Society Transactions*.

[11] Mates J. M., Sanchez-Jimenez F. (1999). Antioxidant enzymes and their implications in pathophysiologic processes. *Frontiers in Bioscience*.

[12] Singal P. K., Petkau A., Gerrard J. M., Hrushovetz S., Foerster J. (1988). Free radicals in health and disease. *Molecular and Cellular Biochemistry*.

[13] Johansen J. S., Harris A. K., Rychly D. J., Ergul A. (2005). Oxidative stress and the use of antioxidants in diabetes: linking basic science to clinical pratice. *Cardiovascular Diabetology*.

[14] Choi K. M., Zhong Y., Hoit B. D., Grupp I. L., Hahn H., Dilly K. W., Guatimosim S., Jonathan Lederer W., Matlib M. A. (2002). Defective intracellular Ca^2+^ signaling contributes to cardiomyopathy in type 1 diabetic rats. *American Journal of Physiology: Heart and Circulatory Physiology*.

[15] Williamson C. L., Dabkowski E. R., Baseler W. A., Croston T. L., Alway S. E., Hollander J. M. (2010). Enhanced apoptotic propensity in diabetic cardiac mitochondria: influence of subcellular spatial location. *American Journal of Physiology—Heart and Circulatory Physiology*.

[16] Koya D., King G. L. (1998). Protein kinase C activation and the development of diabetic complications. *Diabetes*.

[17] Inoguchi T., Sonta T., Tsubouchi H., Etoh T., Kakimoto M., Sonoda N., Sato N., Sekiguchi N., Kobayashi K., Sumimoto H., Utsumi H., Nawata H. (2003). Protein kinase C-dependent increase in reactive oxygen species (ROS) production in vascular tissues of diabetes: role of vascular NAD(P)H oxidase. *Journal of the American Society of Nephrology*.

[18] Nagareddy P. R., Soliman H., Lin G., Rajput P. S., Kumar U., McNeill J. H., MacLeod K. M. (2009). Selective inhibition of protein kinase C *β*2 attenuates inducible nitric oxide synthase-mediated cardiovascular abnormalities in streptozotocin-induced diabetic rats. *Diabetes*.

[19] Xia Z., Kuo K. H., Nagareddy P. R., Wang F., Guo Z., Guo T., Jiang J., McNeill J. H. (2007). N-acetylcysteine attenuates PKC*β*2 overexpression and myocardial hypertrophy in streptozotocin-induced diabetic rats. *Cardiovascular Research*.

[20] Guo Z., Xia Z., Jiang J., McNeill J. H. (2007). Downregulation of NADPH oxidase, antioxidant enzymes, and inflammatory markers in the heart of streptozotocin-induced diabetic rats by *N*-acetyl-L-cysteine. *American Journal of Physiology: Heart and Circulatory Physiology*.

[21] Salvemini D., Wang Z.-Q., Zweier J. L. (1999). A nonpeptidyl mimic of superoxide dismutase with therapeutic activity in rats. *Science*.

[22] Eurich D. T., Majumdar S. R., Tsuyuki R. T., Johnson J. A. (2004). Reduced mortality associated with the use of ACE inhibitors in patients with type 2 diabetes. *Diabetes Care*.

[23] Ye G., Metreveli N. S., Ren J., Epstein P. N. (2003). Metallothionein prevents diabetes-induced deficits in cardiomyocytes by inhibiting reactive oxygen species production. *Diabetes*.

[24] Schindler C. (2008). ACE-inhibitor, AT1-receptor-antagonist, or both? A clinical pharmacologist's perspective after publication of the results of ONTARGET. *Therapeutic Advances in Cardiovascular Disease*.

[25] Cumashi A., Ushakova N. A., Preobrazhenskaya M. E., D'Incecco A., Piccoli A., Totani L., Tinari N., Morozevich G. E., Berman A. E., Bilan M. I., Usov A. I., Ustyuzhanina N. E., Grachev A. A., Sanderson C. J., Kelly M., Rabinovich G. A., Iacobelli S., Nifantiev N. E. (2007). A comparative study of the anti-inflammatory, anticoagulant, antiangiogenic, and antiadhesive activities of nine different fucoidans from brown seaweeds. *Glycobiology*.

[26] Richard B., Bouton M.-C., Loyau S., Lavigne D., Letourneur D., Jandrot-Perrus M., Arocas V. (2006). Modulation of protease nexin-I activity by polysaccharides. *Thrombosis and Haemostasis*.

[27] Chen J., Wang W., Zhang Q., Li F., Lei T., Luo D., Zhou H., Yang B. (2013). Low molecular weight fucoidan against renal ischemia-reperfusion injury via inhibition of the MAPK signaling pathway. *PLoS ONE*.

[28] Yang W., Yu X., Zhang Q., Lu Q., Wang J., Cui W., Zheng Y., Wang X., Luo D. (2013). Attenuation of streptozotocin-induced diabetic retinopathy with low molecular weight fucoidan via inhibition of vascular endothelial growth factor. *Experimental Eye Research*.

[29] Cui W., Zheng Y., Zhang Q., Wang J., Wang L., Yang W., Guo C., Gao W., Wang X., Luo D. (2014). Low-molecular-weight fucoidan protects endothelial function and ameliorates basal hypertension in diabetic Goto-Kakizaki rats. *Laboratory Investigation*.

[30] Fitton J. H. (2011). Therapies from fucoidan; multifunctional marine polymers. *Marine Drugs*.

[31] Zhu Z., Zhang Q., Chen L., Ren S., Xu P., Tang Y., Luo D. (2010). Higher specificity of the activity of low molecular weight fucoidan for thrombin-induced platelet aggregation. *Thrombosis Research*.

[32] Wang J., Zhang Q., Zhang Z., Li Z. (2008). Antioxidant activity of sulfated polysaccharide fractions extracted from *Laminaria japonica*. *International Journal of Biological Macromolecules*.

[33] Livak K. J., Schmittgen T. D. (2001). Analysis of relative gene expression data using real-time quantitative PCR and the 2-ΔΔCT method. *Methods*.

[34] Badenhorst D., Maseko M., Tsotetsi O. J. (2003). Cross-linking influences the impact of quantitative changes in myocardial collagen on cardiac stiffness and remodelling in hypertension in rats. *Cardiovascular Research*.

[35] Cai L., Kang Y. J. (2003). Cell death and diabetic cardiomyopathy. *Cardiovascular Toxicology*.

[36] El-Omar M. M., Yang Z.-K., Phillips A. O., Shah A. M. (2004). Cardiac dysfunction in the Goto-Kakizaki rat—a model of type II diabetes mellitus. *Basic Research in Cardiology*.

[37] Aragno M., Mastrocola R., Alloatti G., Vercellinatto I., Bardini P., Geuna S., Catalano M. G., Danni O., Boccuzzi G. (2008). Oxidative stress triggers cardiac fibrosis in the heart of diabetic rats. *Endocrinology*.

[38] Frustaci A., Kajstura J., Chimenti C., Jakoniuk I., Leri A., Maseri A., Nadal-Ginard B., Anversa P. (2000). Myocardial cell death in human diabetes. *Circulation Research*.

[39] Selvaraju V., Joshi M., Suresh S., Sanchez J. A., Maulik N., Maulik G. (2012). Diabetes, oxidative stress, molecular mechanism, and cardiovascular disease—an overview. *Toxicology Mechanisms and Methods*.

[40] Kim H. W., Cho Y. S., Lee H. R., Park S. Y., Kim Y.-H. (2001). Diabetic alterations in cardiac sarcoplasmic reticulum Ca^2^+-ATPase and phospholamban protein expression. *Life Sciences*.

[41] Kumar S., Kain V., Sitasawad S. L. (2012). High glucose-induced Ca^2+^ overload and oxidative stress contribute to apoptosis of cardiac cells through mitochondrial dependent and independent pathways. *Biochimica et Biophysica Acta: General Subjects*.

[42] Baraka A., AbdelGawad H. (2010). Targeting apoptosis in the heart of streptozotocin-induced diabetic rats. *Journal of Cardiovascular Pharmacology and Therapeutics*.

[43] Huynh K., Kiriazis H., Du X.-J., Love J. E., Gray S. P., Jandeleit-Dahm K. A., McMullen J. R., Ritchie R. H. (2013). Targeting the upregulation of reactive oxygen species subsequent to hyperglycemia prevents type 1 diabetic cardiomyopathy in mice. *Free Radical Biology & Medicine*.

[44] Marnett L. J. (1999). Lipid peroxidation—DNA damage by malondialdehyde. *Mutation Research: Fundamental and Molecular Mechanisms of Mutagenesis*.

[45] Ceriello A. (2003). New insights on oxidative stress and diabetic complications may lead to a “causal” antioxidant therapy. *Diabetes Care*.

[46] Ramakrishna V., Jailkhani R. (2008). Oxidative stress in non-insulin-dependent diabetes mellitus (NIDDM) patients. *Acta Diabetologica*.

[47] Yokota T., Kinugawa S., Hirabayashi K., Matsushima S., Inoue N., Ohta Y., Hamaguchi S., Sobirin M. A., Ono T., Suga T., Kuroda S., Tanaka S., Terasaki F., Okita K., Tsutsui H. (2009). Oxidative stress in skeletal muscle impairs mitochondrial respiration and limits exercise capacity in type 2 diabetic mice. *American Journal of Physiology: Heart and Circulatory Physiology*.

[48] Geraldes P., King G. L. (2010). Activation of protein kinase C isoforms and its impact on diabetic complications. *Circulation Research*.

[49] Soliman H., Gador A., Lu Y.-H., Lin G., Bankar G., MacLeod K. M. (2012). Diabetes-induced increased oxidative stress in cardiomyocytes is sustained by a positive feedback loop involving Rho kinase and PKC*β*2. *American Journal of Physiology—Heart and Circulatory Physiology*.

[50] Li N., Zhang Q., Song J. (2005). Toxicological evaluation of fucoidan extracted from *Laminaria japonica* in Wistar rats. *Food and Chemical Toxicology*.

[51] Myers S. P., O'Connor J., Fitton J. H., Brooks L., Rolfe M., Connellan P., Wohlmuth H., Cheras P. A., Morris C. (2010). A combined phase I and II open label study on the effects of a seaweed extract nutrient complex on osteoarthritis. *Biologics: Targets and Therapy*.

